# Zinc at the Host–Fungus Interface: How to Uptake the Metal?

**DOI:** 10.3390/jof6040305

**Published:** 2020-11-21

**Authors:** Lucas Weba Soares, Alexandre Melo Bailão, Célia Maria de Almeida Soares, Mirelle Garcia Silva Bailão

**Affiliations:** Departamento de Bioquímica e Biologia Molecular, Instituto de Ciências Biológicas, Universidade Federal de Goiás, Goiânia 74690-900, Goiás, Brazil; weba-soares@hotmail.com (L.W.S.); ambailao@ufg.br (A.M.B.); cmasoares@gmail.com (C.M.d.A.S.)

**Keywords:** zinc homeostasis, fungal pathogenesis, ZIP transporters

## Abstract

Zinc is an essential nutrient for all living organisms. However, firm regulation must be maintained since micronutrients also can be toxic in high concentrations. This notion is reinforced when we look at mechanisms deployed by our immune system, such as the use of chelators or membrane transporters that capture zinc, when threatened with pathogens, like fungi. Pathogenic fungi, on the other hand, also make use of a variety of transporters and specialized zinc captors to survive these changes. In this review, we sought to explain the mechanisms, grounded in experimental analysis and described to date, utilized by pathogenic fungi to maintain optimal zinc levels.

## 1. Introduction

Among the metals of major importance for maintenance of basic activities in an organism, zinc is the second most abundant concerning the association with enzymes with known structures [1] as well as in total cellular distribution, surpassed only by iron [2]. This metal is known especially for its structural role in the regulation of gene transcription by the zinc fingers, considered as the largest group of transcription regulators [3,4,5]. In fungi, zinc binding to transcription factors and other proteins is quite similar, ranging from approximately 9% of the proteome in *Aspergillus fumigatus* and 7.5% in *Saccharomyces cerevisiae* [6]. Zinc relevance is further stressed by our immune system that, when confronted by a fungal pathogen, often deploys an array of mechanisms in order to manipulate microbial access to the metal. Neutrophils release extracellular traps to restrict extracellular zinc availability via calprotectin, a zinc binding protein, released at inflammatory sites [7]. Zinc limitation is also observed intracellularly when infected macrophages restrict fungal growth by pumping the metal out of the phagosome [8]. To ensure survival, pathogenic fungi express an array of zinc transporters with variable degrees of affinity to zinc and, thus, maintain growth in harsh conditions [9,10,11,12].

Progressive studies have been published in order to understand how those transporters work in pathogenic fungi, unveiling two major groups of zinc transporters: the Zrt- and Irt-like Protein (ZIP) and the Cation Diffusion Facilitator (CDF) families of transporters [13,14]. The ZIP family consists of membrane proteins responsible for the transport of zinc into the cytosol, either from the extracellular environment [9] or from intracellular membrane compartments, such as the vacuole or endoplasmic reticulum [15]. The CDF family transports zinc from the cytosol to organelles, maintaining essential zinc-depending metabolic processes [16] as well as quickly reducing zinc cytosolic levels in case of “zinc shock” [14]. All the main proteins of both families are tightly regulated by a single transcription factor, capable of sensing minor alterations in cytosolic zinc levels [17,18,19]. In this review, we sought to present the overall knowledge concerning zinc homeostasis in human pathogenic fungi, focusing on genes and proteins experimentally proven to be involved in Zn transport as well as zinc uptake regulation.

## 2. Current Knowledge on Zinc Homeostasis in Pathogenic Fungi

Our first goal was to obtain a complete landscape of the actual understanding regarding zinc homeostasis by gathering a list of all pathogenic fungi known to date. Literature classifies approximately 300 fungal species as pathogenic to humans [20,21]. When searching for articles concerning zinc homeostasis in public available databases, only 13 species have a minimum of one study focused on the understanding zinc homeostasis (Table 1). After analyzing the acquired data, we were able to divide those species into two distinct mechanisms of zinc uptake regulation: those that are zinc-dependent, and those that are zinc/pH-dependent for the transcription of their main zinc transporters. Most of fungi lack sufficient studies that either proper characterize the function of the predicted transporters or stand with not enough experiments to properly classify them. Those include *Paracoccidioides brasiliensis, Paracoccidioides lutzii, Emmonsia parva, Emmonsia crescens, Trichophyton mentagrophytes, Histoplasma capsulatum*, and *Candida dubliniensis*. Members of *Paracoccidioides* and *Emmonsia* genera will not be discussed here since the predicted transporters lack functional confirmatory experiments [22,23,24,25]. *T. mentagrophytes, H. capsulatum*, and *C. dubliniensis* published data only described the function of one protein involved in zinc homeostasis, which will be slightly discussed further on. It is important to point out that the lack of pH-dependent regulation by fungi that currently have the zinc homeostasis mechanisms solely relied on Zn availability (“Not enough data” in Table 1) may be attributed to the lack of research on the topic. Additionally, classification of zinc uptake in *Cryptococcus neoformans* as zinc dependent was based on the representative of the same genus, *Cryptococcus gattii*, and as reviewed by Gerwien and co-workers [26]. Similarly, *C. dubliniesis* was classified as *Candida albicans* regarding pH regulation of zinc uptake.

## 3. Zinc-Dependent Zinc Uptake

### 3.1. Saccharomyces cerevisiae

The initial studies concerning zinc homeostasis began with *S. cerevisiae* [27]. Although not notorious for its pathogenic capabilities, infections have been reported in several immunocompromised individuals [28,29,30]. Furthermore, the baker’s yeast is also the target of most articles regarding zinc homeostasis, serving as a starting point on subsequent studies of other pathogenic fungi.

The first reported gene related to zinc transport in *S. cerevisiae* was the zinc resistance conferring (*ZRC1*) [27]. Initially recognized as the name implies, being essential for survival under conditions of high zinc availability, *ZRC1* was later shown to be responsible for the transport of zinc from the cytoplasm into the vacuole [31]. In such a way, it protects the cell from “zinc shock”, a term applied whenever a cell receives a burst of zinc in a short amount of time [32]. However, even before the specificity of Zrc1 was demonstrated, scientists were already unveiling the regulatory mechanism that manipulates not only Zrc1, but the zinc transporters in general: the zinc-responsive activator protein, Zap1 [33].

*ZAP1* is a gene that encodes an 880 amino acids protein of the same name, involved not only in the transcriptional regulation of the main zinc transporters [17], but also in its own expression and in the regulation of approximately 80 genes on *S. cerevisiae* genome [34]. All of these genes are predicted to play a role, directly or indirectly, on the cell’s response to variations in zinc levels. Zap1 regulates its target genes by binding to specific sequences on the promoter region, namely the zinc responsive elements (ZREs) [35]. Whereas initial studies characterized the presence of five zinc fingers domains in Zap1 [33], it was later shown that the number is greater than previously described [36]. Indeed, Zap1 has seven zinc fingers domains, five of which retain protein–DNA interaction properties, binding to ZREs. The remaining two are clustered in an acidic activation domain 2 (AD2) and perform interactions with zinc ions in a regulatory fashion, rather than the conventional structural function [37]. Soon after that, a regulatory mechanism was also observed in activation domain 1 (AD1) with no predicted zinc finger domains, but able to bind to zinc ions due to a high concentration of histidine and cysteine residues [38]. Individually, these domains constitute a highly sensitive zinc sensing system, with predicted protein fold changes based on zinc availability on the nucleus and, hence, in the cell [36,39]. These changes, in turn, are necessary in order to recruit other regulatory co-activators that promote expression of a number of genes [40].

**Figure 1:**
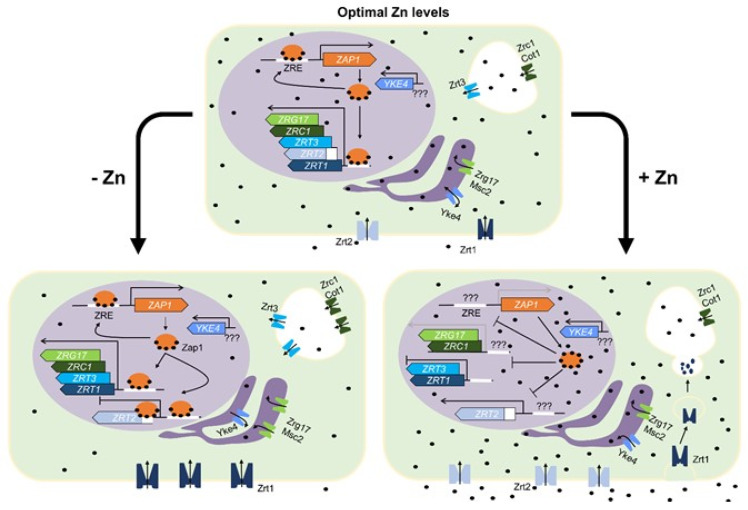
Zinc homeostasis mechanisms in *Saccharomyces cerevisiae*. Zinc is represented by black dots. Proteins that transport zinc to the cytosol as well as the correspondent genes are represented by different shades of blue; intracellular zinc transporters and genes are represented by shades of green. At optimal zinc levels, Zap1 (orange circle) induces itself and all transporters at different levels with the exception of Yke4 (regulation currently unknown), Msc2, and Cot1 (not Zap1-dependent). Under zinc deprivation conditions, Zap1 induces itself as well as *ZRG17*, *ZRC1*, *ZRT3,* and *ZRT1* genes while repressing *ZRT2* by binding to the zinc responsive elements (ZRE) near *ZRT2* TATA box (white box upstream *ZRT2*). Zrt1 and Zrt2 are cytoplasmatic membrane transporters, Zrc1/Cot1 and Zrt3 are vacuolar transporters and Zrg17 is part of a complex with Msc2 in the ER. Under excess zinc conditions, Zap1 is inactive and unable to bind to ZREs on target genes. However, *ZRG17* and *ZRC1* are expressed at reduced levels. *ZRT3* and *ZRT1* are repressed and *ZRT2* is induced by yet to be described mechanisms. Zrt1 is also quickly internalized and degraded in this condition.

Not only the sensing provided by the multiple zinc fingers domains in Zap1 is used by the cell to regulate gene transcription. The ZREs (consensus 5′-ACCTTNAAGGT-3′) also play a key role in the regulation of target genes based on (i) the binding affinity to Zap1, (ii) number of copies, and (iii) location in the promoter region [17,41]. Zap1 binds to ZRE with low, high, or moderate affinity, directly influencing transcription. Based on current literature, we are able to predict a pattern of expression of Zap1 and its target genes in three different scenarios: optimal, low, and excessive zinc conditions (Figure 1). Under optimal zinc levels, Zap1 generally induces its own expression as well as that of all transporters regulated by it to various degrees. These transporters are members of the ZIP family, such as *ZRT1* (the high affinity transporter [9]), *ZRT2* (the low affinity transporter [42]), and *ZRT3* (the vacuolar zinc exporter [15]), and also proteins that belong to the CDF family, like *ZRG17* and *ZRC1*, the zinc importers of the endoplasmic reticulum (ER) and vacuole, respectively [43,44]. A few transporters not regulated by Zap1 help in the maintenance of stable zinc levels inside the cell. *MSC2* is associated with *ZRG17* in a complementary manner. Similarly, *COT1*, a cobalt vacuole transporter, complements *ZRC1* function [44]. Lastly, *YKE4*, a member of the ZIP family of transporters, was showed to complement the function of Zrg17/Msc2 in the ER in a bidirectional fashion, transporting zinc inside or out of this organelle based on necessity [45]. However, it is rather unclear if this transporter is regulated by Zap1 and/or zinc.

On a model of zinc scarcity, Zap1 induces its own expression, increasing in quantity [17]. Such increase comes with availability to bind to low affinity ZRE regions, such as the one found near the TATA box of the *ZRT2* gene, physically blocking its transcription [41]. This happens since Zrt2 presents low affinity to zinc, therefore creating unnecessary energy costs if expressed in a zinc deprivation environment [42]. In such conditions, *S. cerevisiae* induces the expression of a high affinity transporter. To do so, Zap1 also binds to ZREs found in the promoter region of the *ZRT1* gene [9]. In accordance, Zrt3, the zinc efflux transporter found in the vacuole, is also induced, allowing usage of the zinc stored in this organelle [15].

Following this logic, it would be expected that, during zinc deprivation, the cell would reduce the expression of transporters that move zinc from the cytosol into different intracellular compartments. In fact, that is not exactly the case. When in zinc scarcity, both Zrc1 and Zrg17, the CDFs found in the vacuole and ER, respectively, are induced [43,46]. This contradiction is supported by two theories: (i) repression of *ZRC1* would render the cell defenseless to “zinc shock” as well as deprive vacuolar enzymes of this important metal [31,44]; (ii) a diversity of metabolic pathways takes place in the ER and some of them require zinc to function. Thus, overall cell health would be compromised by severely reducing the expression of the zinc transporter complex Zrg17/Msc2 [43,47] as well as Zrc1/Cot1. It is important to note that *MSC2* and *COT1* are not regulated by Zap1 [34,48,49]. *MSC2* regulation in particular is suggested to occur at a post-transcriptional level [49]. Finally, under zinc deprivation, the ER transporter Yke4 exports zinc out of the organelle [45]. 

In a high zinc availability environment, *ZRC1* and *ZRG17* are expressed at lower levels (Figure 1) [43,44]. As a matter of fact, the expression of most of Zap1 regulated genes is reduced to different degrees in a zinc replete environment [9,15,17,43,44], *ZRT2* being the sole exception. Initially, it has been proposed that as zinc levels increase, Zap1 no longer binds to the low affinity ZRE of *ZRT2* and only binds to the high affinity ZREs, which in turn results in *ZRT2* induction [41]. However, it has later been shown that this was not the case. Once zinc levels are high, Zap1 is inactivated and unable to bind to ZREs [50]. Curiously, ZRE sites are untouched by Zap1 when zinc availability is high, regardless of Zap1′s quantity or location inside the cell, suggesting a yet to be characterized process of *ZRT2* induction. Lastly, the necessity of a rapid adaptation during the transition of a depleted to replete zinc condition is accomplished by a shift between Zrt1 and Zrt2 [42], which it is not achieved solely by changes in gene expression. Additionally, *S. cerevisiae* resorts to ubiquitination of Zrt1, followed by its endocytosis and degradation in response to zinc excess [51]. In the context of zinc excess, Yke4 transports the metal into the ER [45].

### 3.2. Cryptococcus neoformans and Cryptococcus gattii

Although it does not harbor many pathogenic species, the *Cryptococcus* genus gained even more coverage after an outbreak of cryptococcosis in immunocompetent individuals [52,53]. Out of four major *Cryptococcus* pathogenic species [54], *C. neoformans* and *C. gattii* are the ones with published data on zinc homeostasis described to date [11,55,56,57]. Those species harvest zinc from the environment in a very similar fashion. *C. gattii* harbors a Zap1 ortholog whose activity regulates a variety of genes related to the adaptation to zinc scarcity and that is required for full virulence in a murine model of infection [55].

The main zinc transporters in both *C. gattii* and *C. neoformans* are Zip1 and Zip2 [11,56]. A functional redundancy has been proposed for those two proteins in *C. gattii*. Although zinc uptake is mainly attributed to Zip1 in this species, murine survival assays with mutant strains did not point out which transporter is mostly related to zinc acquisition inside the host. Indeed, percentage survival of mice infected with *zip1Δ* or *zip2Δ* was similar. A double mutant, however, presented a severally reduced virulence. These findings may be explained by compensatory machinery, not conserved in fungi, in which the absence of *ZIP2* leads to the upregulation of *ZIP1* [11].

Suchlike analysis was also conducted with *C. neoformans*, providing different results. The *C. neoformans zip1Δ* strain showed reduced virulence in mice when compared to both *zip2Δ* and wild type strains. The authors speculate that such difference may be coupled with infection sites. While *C. neoformans* main site is the brain, *C. gattii* is more often found in the lungs of immunocompetent patients. Although *ZIP2* seems to play a minor role in virulence, no evidence was found regarding its function as critical for zinc transport in vitro [56]. The double mutant strains *zip1Δzip2Δ* of both *C. gattii* and *C. neoformans* are able to grow upon zinc supplementation, suggesting an additional and yet to be characterized zinc uptake mechanism [11,56]. Besides *ZIP1* and *ZIP2*, the ZIP family gene *ZIP3* was also found to be present in *C. gattii* genome. Despite being regulated by zinc levels, *ZIP3* gene encodes a protein involved in manganese transport in the Golgi apparatus. Zip3 was shown to play a role in reactive oxygen species sensitivity and other virulence aspects in *C. gattii*, such as melanin deposition and secretion of glucuronoxylomannan [58]. Finally, a recent investigation described the Zrc role in zinc detoxification in *C. neoformans*, just as that performed by *S. cerevisiae* Zrc1. The study also evidenced that Zrc1 is not required for virulence in vivo [57], an expected result given that it has been previously shown that macrophages promote zinc deprivation rather than intoxication during *C. neoformans* infection [59].

## 4. Zinc- and pH-Dependent Zinc Uptake

### 4.1. Aspergillus fumigatus

The *Aspergillus* genus consists of approximately 180 species with distinct characteristics and properties, often used for commercial purposes and biological studies of fungal reproductive patterns. Some species, however, cause a range of pathologies called aspergillosis, which mainly affect the respiratory tract of immunocompromised individuals [60]. Lung tissue is an alkaline environment, where zinc availability is low. It happens because Zn^2+^ ions tend to form insoluble metallic complexes as pH increases [61]. Additionally, at physiological pH most zinc is bound to host proteins intra- and extracellularly [62], which makes the amount of the readily available metal very low. Therefore, *Aspergillus* must make use of several mechanisms to keep up with adequate metal levels.

**Figure 2:**
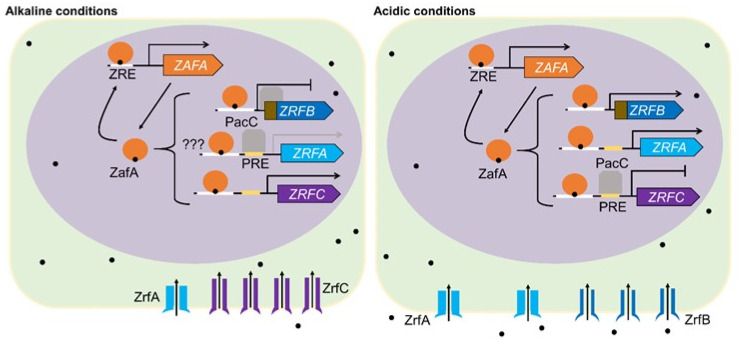
Zinc- and pH-dependent zinc homeostasis mechanisms in *Aspergillus fumigatus*. Zinc is represented by black dots. Proteins and genes involved in zinc transport are represented by shades of blue, with ZrfC in purple. In conditions of zinc deprivation at acid pH, ZafA (orange circle) induces *zrfA* and *zrfB* as well as its own expression by binding to the ZREs (white line) in the promoter region of the target genes. ZafA also binds to *zrfC’s* ZRE. However, PacC (grey rectangle) is believed to interact negatively with ZafA, since both PacC binding site (PRE, yellow line) and ZRE are in proximity, thereby repressing *zrfC* transcription. In conditions of zinc deprivation at alkaline pH, ZafA induces *zrfC* expression and PacC represses *zrfB* by binding to a PRE region near the TATA box of the gene (brown box). PacC also reduces *zrfA* expression by unknown mechanisms.

The pathogenic species *A. fumigatus* encodes a transcription factor, ZafA, which modulates the response to zinc starvation. ZafA also self-regulates by binding to the ZRE sequence on its own promoter, similarly to Zap1 in *S. cerevisiae* [19,63]. Among the eight transporters of the ZIP family encoded by *A. fumigatus*, three were characterized in experimental analyzes and are involved in zinc uptake under acid or alkaline conditions (*zrfA, zrfB,* and *zrfC*), four are ZafA targets (*zrfA, zrfB, zrfC,* and *zrfF*), and five are predicted to play a role in zinc homeostasis (*zrfD, zrfE, zrfF, zrfG,* and *zrfH*). As functional characterization of the predicted transporters is still unavailable, they will not be discussed here [10,64,65,66,67]. Based on those studies, it is possible to outline the behavior of *A. fumigatus* in two situations: zinc deprivation (i) in acidic and (ii) in alkaline conditions (Figure 2).

In addition to ZafA, adaptation to zinc limiting conditions in *A. fumigatus* is also modulated by PacC, a transcription factor responsive to the ambient pH, which regulates its target genes by binding to the pH-responsive elements (PREs) at the promoter region [68]. Under acidic zinc deprivation conditions, both *zrfA* and *zrfB* are induced by ZafA [63], whilst *zrfC* is repressed, presumably by yet undefined physical interaction between ZafA and PacC, given the proximity of the binding sites at target promoters. In such scenario, a negative interference of PacC over ZafA activity is believed to occur [66]. In neutral-alkaline pH *pacC* expression is slightly higher compared to acidic conditions, regardless of zinc availability. Under neutral-alkaline, zinc-limiting conditions this transcription factor binds to the PRE site near the TATA box of *zrfB*, completely inhibiting the expression of this transporter, similarly to *ZRT2* regulation by Zap1 in *S. cerevisiae* (Figure 1). The PRE site upstream *zrfA*, on the other hand, is situated within one of the three ZRE sites in its promoter region. As a consequence, *zrfA* is not completely repressed by PacC [10]. Unlike what happens in acidic environment, *zrfC* is required for zinc uptake under alkaline, zinc limiting conditions. Curiously, PacC only harbors a negative role over the expression of *zrfC*, whose induction is strictly related to ZafA [66]. How and why PacC is not involved in *zrfC* induction even with multiple PRE sites at the promoter region of *zrfC* are questions that remain unanswered. In summary, under zinc-limiting conditions, ZafA induces the expression of the ZIP transporters, regardless of the pH. Otherwise, PacC appears to interfere in some way with ZafA function [66], resulting in the repression of *zrfA, zrfB* and *zrfC*, the first two in alkaline medium and the last in acidic conditions.

*Aspergillus* species are metabolically versatile, presenting similar growth rates in a vast range of pH values [69]. The same is not true for *S. cerevisiae*, which grows optimally in acidic conditions [70]. Therefore, it is not surprising the fact that ambient pH influences zinc homeostasis in *A. fumigatus* in a way not seen in *S. cerevisiae*. Indeed, expression of *ZRT1* and *ZRT2* does not change when yeast is exposed to high pH values [71,72,73], which indicates that Zrt1 and Zrt2 uptake zinc required by the fungus to grow in both acidic and alkaline media. In contrast, *A. fumigatus* uses ZrfC, with no ortholog in *S. cerevisiae*, to acquire zinc in alkaline zinc-limiting conditions. This transporter presents zinc-binding motifs on its N-terminus, absent in ZrfA and ZrfB, and is necessary for zinc uptake in lung tissue [65,66].

The promoter region of *zrfC* is shared with *aspf2*, which encodes an allergen of *A. fumigatus* [74]. *Aspf2*, like *zrfC*, is induced under alkaline zinc-limiting conditions on dependence of ZafA. Even though molecular mechanisms describing the role of Asp2 in zinc uptake are unavailable, this protein was shown to be necessary for fungal growth in extreme zinc-limiting conditions [66]. The involvement of Aspf2 on zinc acquisition is further reinforced by the fact this antigen is produced exclusively in conditions of low zinc availability [75] and is supposedly a zinc binding protein [66].

A recent study has shown that the transition metal iron as well as a transcription factor related to copper tolerance also influence zinc homeostasis in *A. fumigatus* [76,77]. Cai and co-workers demonstrated that Ace1, previously described as a copper detoxifying transcriptional regulator, is also essential for adaptation to high zinc levels. This is accomplished mainly by the Ace1 dependent expression of *zrcA*, a *S. cerevisiae ZRC1* ortholog [77]. Additionally, *A. fumigatus* growth in zinc-replete conditions relies on iron availability. The expression of different transcription subunits of ZafA is controlled by iron levels, culminating in basal amounts of this transcription factor that regulate optimal fungal growth in zinc-replete conditions [76]. Those examples highlight the complexity and tight regulation of zinc homeostasis performed by *A. fumigatus* in response to pH, zinc and iron levels, as well as the interplay between transition metals metabolism.

### 4.2. Candida albicans and Candida dubliniensis

The genus *Candida* is well established in literature due to its ability to promote disease in the mucosa, blood, cutaneous tissue, and internal organs. Among the more than 15 pathogenic species belonging to the genus *Candida*, *C. albicans* is responsible for most cases of candidiasis. Despite the increase in infections caused by *C. parapsilosis* and *C. glabrata, C. albicans* is the main causative agent related to candidemia [78], and also responsible for 95% of oral candidiasis cases [79]. *C. dubliniensis* in counterpart, is considered less pathogenic [80] and is mainly associated with HIV-infected patients [81].

**Figure 3:**
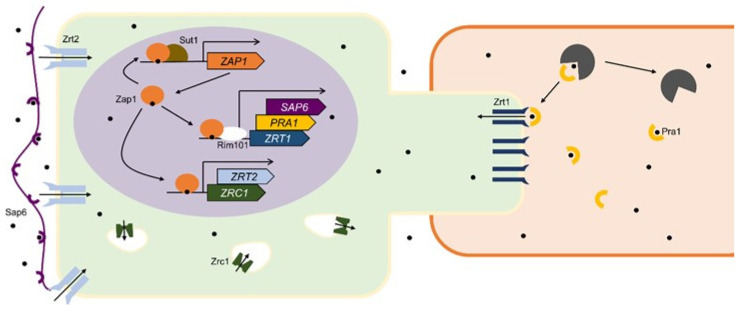
Mechanisms utilized in the maintenance of zinc homeostasis in *Candida albicans* under zinc deprivation. Zap1 is induced by Sut1 and induces *ZRT2* (light blue) and *ZRC1* (green), a plasma membrane zinc transporter and a zincosome zinc importer, respectively. Coupled with Rim101 (white), Zap1 also induces *SAP6*, *PRA1,* and *ZRT1*. Sap6 (purple) is a biofilm formation protein that also functions as a zinc “magnet”, facilitating metal internalization. Zrt1 (dark blue) is a zinc plasma membrane transporter that harvests zinc obtained by Pra1 (yellow), a secreted protein capable of detaching zinc bound to other proteins (represented in black) inside infected cells.

The mechanisms used by *C. albicans* to acquire zinc in an environment deprived of the metal are shown in Figure 3. Unlike *S. cerevisiae*, Zap1 of *C. albicans*, named Csr1, has seven zinc fingers, with no ADs identified thus far [18,82]. *C. albicans* Zap1 also binds to its own promoter and regulates itself [18], a characteristic apparently conserved in fungi. Functional analysis of transcriptional regulators at different stages of infection demonstrated an associative effect between Zap1 and Sut1, a homolog of a zinc cluster regulator involved in sterol uptake in *S. cerevisiae* [83]. Virulence of the attenuated *sut1∆/∆* mutant was fully restored following ectopic expression of *ZAP1* in such strain, demonstrating that *ZAP1* expression during infection is dependent on Sut1 [84].

As in *A. fumigatus*, the adaptation to zinc scarcity in *C. albicans* relies on metal uptake systems that differ according to the pH. In neutral-alkaline conditions, the fungus secretes Pra1, a protein with zinc binding properties, ortholog of Aspf2 [85], which captures extracellular zinc and is able to reassociate to the cell surface afterwards. This “zincophore” system, named in analogy to the iron scavengers siderophores, also includes Zrt1, a plasma membrane ZIP transporter that receives zinc bound to Pra1 [86]. Both Zrt1 and Pra1 share the same promoter and are regulated by the transcription factors Zap1 and Rim101, which mediate responses to zinc availability and environmental pH, respectively [86,87,88]. Rim101 is an ortholog of *A. fumigatus* PacC and is required for pathogenesis [88,89,90]. Additionally, both Zap1 and Rim101 support filamentation in *C. albicans*, linking the dimorphism process with zinc homeostasis and pH [82,91].

Zinc uptake in *C. albicans* under acidic conditions is mediated by Zrt2, which is also able to support growth in alkaline zinc limiting environment, being considered the major zinc importer in this fungus [92]. *ZRT2* is regulated by pH [88,92], Rim101 [93], zinc [92], and Zap1 [87,94]. Crawford and coworkers sought to clarify several aspects related to proteins responsible for the zinc flux into *C. albicans* organelles. Using bioinformatics tools in conjunction with experimental analyses, they were able to demonstrate the activity of Zrc1. This transporter, previously known to mediate the entry of zinc into the vacuole in *S. cerevisiae*, is not associated with the vacuole membrane in *C. albicans*. Instead, Zrc1 is a zincosomal importer. Despite the localization difference, the function of Zrc1 in *C. albicans* is similar to that in *S. cerevisiae*: to ensure the flow of zinc into an intracellular compartment when the cell is exposed to toxic levels of the metal [92]. In agreement, Zrc1 was found to be essential for liver colonization, since hepatocytes increase zinc uptake upon infection [95].

Finally, a recent paper gave new insights into *C. albicans* zinc metabolism by studying a considerably well-characterized protein, the aspartic proteinase Sap6. It is an alkaline pH induced protein, involved in cellular aggregation and whose expression is induced during oropharyngeal candidiasis, along with genes involved in zinc uptake (*ZRT1, ZRT2,* and *PRA1*) [88,94,96,97]. Sap6 belongs to Zap1 and Rim101 regulons [84,87] and supports zinc acquisition by binding to the metal for posterior internalization by membrane transporters [97].

Initial understanding of zinc homeostasis in *C. dubliniensis*, a less threatening *Candida* species, was provided by Böttcher and coworkers. Researchers generated a *C. dubliniensis CRS1∆/∆* mutant strain and evaluated the effects of gene deletion on virulence as well as the influence of Crs1 on the expression of ZIP orthologs and Pra1. The results showed a pattern similar to that found in *C. albicans*, in which Zrt2 is supposed to be the high-affinity zinc transporter, while Zrt1 seems to play a secondary hole in zinc transport. Moreover, Crs1 was shown to be necessary for growth under zinc-limited conditions and essential for *C. dubliniensis* virulence [18].

## 5. Zinc Uptake Not Related to Depend on pH Regulation Thus Far

### Blastomyces dermatitidis, Histoplasma capsulatum, and Trichophyton mentagrophytes

Studies regarding the mechanisms involved in zinc homeostasis in the genera *Blastomyces, Histoplasma,* and *Trichophyton* are scarce. However, the information obtained so far demonstrates similarities to those mechanisms previously described [25,98,99,100]. Analysis of gene expression in a mouse model of infection revealed the induction of genes orthologs to *ZAP1, ZRT1, ZRT2,* and *PRA1* in *B. dermatitidis* [25]. Recently, Kujoth and coworkers demonstrated a decreased fungal burden of *pra1∆* and *zrt1∆* strains, generated by a CRISPR/Cas9 based gene disruption approach, in a mouse lung infection model when compared to the wild type strain [99]. This evidences the relevance of zinc in mycoses caused by dimorphic fungi.

Contrary to *B. dermatitidis* and *C. albicans*, no *PRA1* ortholog was found in the genus *Histoplasma* [101]. However, when comparing sequences of the ZIP family with the genome of *H. capsulatum*, researchers were able to identify a putative zinc transporter, named Zrt2 [98]. When exposed to a zinc deprivation environment, *H. capsulatum* induces *ZRT2*, which is essential for virulence, as demonstrated by a mouse infection model [98]. Zrt2 also appears to be the high affinity zinc transporter in *H. capsulatum*, similar to Zrt2 in *C. albicans*.

Lastly, we have the most recently described zinc related transcription factor in a pathogenic fungus. Using transcriptomic analysis followed by gene knockout, Zhang and coworkers were able to determine the ZafA ortholog in *T. mentagrophytes*. The transcription factor is essential for growth under zinc-limited conditions and is also important for conidiation. Additionally, possible *zrfA, zrfB,* and *zrfC* orthologs were also found, but functional analyses are still unavailable [100].

## 6. Conclusions

**Figure 4:**
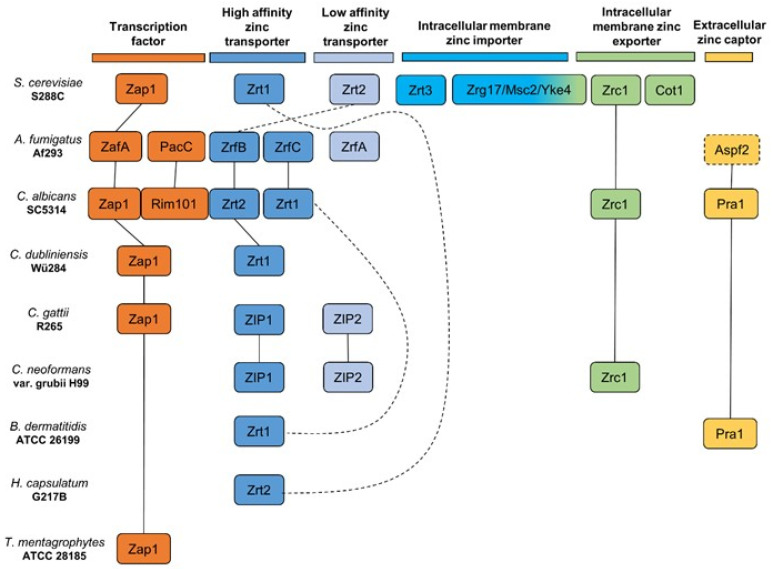
Schematic with all proteins involved in zinc transport, and its regulation, experimentally characterized in pathogenic fungi. Straight lines represent orthologs proven by experimental analysis. Dotted lines represent orthologs consensus based on reviewed studies with pending experimental analysis.

The knowledge about zinc homeostasis in pathogenic fungi has grown considerably over the past few years. Even so, multiple mechanisms remain elusive. Such field is exciting and full of promises given that many of the proteins described are essential for virulence and, in some cases, also hold few similarities to known human proteins, being considered potential targets for novel drugs. However, the rapid and interchangeable development in the field has also made it confusing to newcomers as well as a burden for researchers in the area. During the production of this article, we saw ourselves questioning some classification choices made in certain experimental articles. Some minor, such as Pra1/Aspf2 (pH regulated antigen and *A. fumigatus* allergen) still retaining the same nomenclature given when function was not yet clear. Others concerning, as naming the ZrfC ortholog as Zrt1 in *C. albicans*. As previously mentioned, Zrt1 was first described in *S. cerevisiae* and is expressed in different conditions as those of ZrfC. Thus, giving both, *C. albicans* and *S. cerevisiae*, the same gene name can cause confusion. Lastly, it was consensual that Zrt1 and Zrt2 are named as the high and low affinity zinc transporters, respectively, as initially dictated by *S. cerevisiae* studies. However, in some organisms, such as *C. albicans* and *H. capsulatum*, Zrt2 was designated as the high affinity/major transporter, further blurring pre-established consensus. To simplify and resume current findings, we generated a schematic with all experimentally characterized proteins involved in zinc transport, and its regulation, in pathogenic fungi (Figure 4). It is important to stress that orthologs found in some fungi, like *PRA1* in *C. dubliniensis* and members of the ZIP transporters family (*zrfD, zrfE, zrfF, zrfG,* and *zrfH*) in *A. fumigatus*, were not included in Figure 4 since they have not been experimentally characterized. *Aspf2* was the sole exception given that some experimental analyses have been performed and strongly suggested its role as a *PRA1* ortholog. We hope that changes such as the one given to Zap1 in *C. albicans* (initially named Crs1) can be employed gradually. By facilitating our scientific discoveries didactics, we strive for a broader spectrum of viewers what consequently increases interest publications in the field. As a result, it aids into providing the population with faster and efficient solutions for the growing threat of diseases caused by pathogenic fungi.

## Figures and Tables

**Table 1:** Pathogenic fungal species with published studies on zinc homeostasis.

Zinc Dependent	Zinc and pH Dependent	Not Enough Data ^1^
*Saccharomyces cerevisiae*	*Aspergillus fumigatus*	*Paracoccidioides brasiliensis*
*Cryptococcus gattii*	*Candida albicans*	*Paracoccidioides lutzii*
*Cryptococcus neoformans*	*Candida dubliniensis*	*Emmonsia parva*
		*Emmonsia crescens*
		*Trichophyton mentagrophytes*
		*Histoplasma capsulatum*
		*Blastomyces dermatitidis*

^1^ Regarding influence of pH on zinc uptake.
